# Sclerotherapy or Banding for Oesophageal Varices?

**DOI:** 10.1155/1998/65078

**Published:** 1998-12

**Authors:** Joseph J. Y. Sung

**Affiliations:** Department of Medicine Prince of Wales Hospital The Chinese University of Hong Kong Shatin, Hong Kong China


use of a surgical technique which could not adequately decompress the common channel (supraduodenal exploration with insertion of a T-tube).

The weight of evidence from three previous trials support the use of emergency ES in patients with severe acute pancreatitis due to gallstones irrespective of concomitant acute cholangitis or obstructive jaundice alone. This requires ES to be performed by properly trained personnel [7] and can only be of value if there is a high standard of overall management [1-6].

### References

[1] Neoptolemos J. P., London, N. J., James, D., Carr-Locke, D. L., Bailey, I. A. and Fossard, D. P. (1988). Controlled trial of urgent endoscopic retrograde cholangiopancreatography and endoscopic sphincterotomy versus conservative treatment for acute pancreatitis due to gallstones. *Lancet*, **2**, 979-83.

[2] Nowak, A., Nowakowska-Dulaw, E., Marek, T. A. and Rybicka, J. (1995). Final results of the prospective, randomised, controlled study on endoscopic sphincterotomy versus conventional treatment in acute biliary pancreatitis. *Gastroenterology*, **108**, A380 (Abstract).

[3] Fan, S. T., Lai, C. S., Mok, F. P. T., Lo, C. M., Rheng, S. S. and Wong, J. (1993). Early treatment of acute biliary pancreatitis by endoscopic papaillotomy. *New England Journal of Medicine*, **328**, 228-32.

[4] Luiten, E. J. T., Hop, W. L. J., Lange, J. F. and Browning, H. A. (1995). Controlled clinical trial of selective decontamination for treatment of severe acute pancreatitis. *Ann. Surg.*, **222**, 57-65.

[5] Sainio, V., Kempainen, E., Paolakkainen, P., Taavitsainen, M., Kivisaari, L., Valtonen, V., Haapiainen, R., Schrder, T. and Kivilaakso, E. (1995). Early antibiotic treatment in acute necrotising pancreatitis. *Lancet*, **346**, 663-67.

[6] Kelly, T. R. and Wagner, D. S. (1988). Gallstone pancreatitis: a prospective randomised trial of the timing of surgery. *Surgery*, **104**, 600-5.

[7] Freeman, M. L., Nelson, D. B., Sherman, S. *et al.* (1996). Complications of endoscopic biliary sphicterotomy. *N. Engl. J. Med. C.*, **335**, 909-18.

Margaret D Finch and
Professor John P Neoptolemos
Department of Surgery
University of Liverpool
5th Floor UCD Building
Daulby Street
Liverpool L69 3GA
United Kingdom

### Sclerotherapy or Banding for Oesophageal Varices?

### ABSTRACT

*Sarin, S. K., Govil, A., Jain, A. K., Guptan, R. C., Issar, S. K., Jain, M. and Murthy, N. S. (1997) Prospective randomised trial of endoscopic sclerotherapy versus variceal band ligation for esophageal varices: influence on gastropathy, gastric varices and variceal recurrence. Journal of Hepatology, 26, 826-832.*

*Background/Aims:* **Endoscopic variceal ligation and endoscopic sclerotherapy are both recommended for the prevention of variceal rebleeding. To compare their prevention of variceal rebleeding. To compare their efficacy, their influence on gastric varices and the development of portal gastropathy, 95 patients with variceal bleeding were studied.**
*Methods:* **The patients were randomized to receive weekly endoscopic sclerotherapy using alcohol ($n=48$) or endoscopic variceal ligation ($n=47$). The endoscopic sclerotherapy and endoscopic variceal ligation groups were comparable in etiology, severity of liver disease and grade of varices.**
*Results:* **In the arrest of acute bleed, endoscopic sclerotherapy and endoscopic variceal ligation were comparable (86% vs. 80%, $p=$ ns). Endoscopic variceal ligation as compared to endoscopic sclerotherapy, obliterated esophageal varices in fewer sessions (4.1±1.2 vs. 5.2±1.8, $p<0.01$) and a shorter time (4.4±1.3 vs. 6.9±3.4 wk, $p<0.01$). Three (6.4%) patients bled after endoscopic variceal ligation and 10 (20.8%) after endoscopic sclerotherapy ($p<0.05$). The actuarial percentage of variceal recurrence during a follow-up of 8.5±4.4 months, was higher after endoscopic variceal ligation than endoscopic sclerotherapy (28.7% vs 7.5%, $p<0.05$). Esophageal stricture formation after endoscopic sclerotherapy occurred in five (10.4%) patients, but in none after endoscopic variceal ligation. Significantly more patients developed gastropathy after endoscopic sclerotherapy than ligation (20.5% vs. 2.3%; $p=0.02$). Endoscopic sclerotherapy (52%) and endoscopic variceal ligation (59%) were equally effective in obliterating the lesser curve gastric varices. Six patients died: three in each group.**
*Conclusion:* **(i) Endoscopic sclerotherapy and endoscopic variceal ligation were equally effective in controlling acute bleed; (ii) endoscopic ligation achieved variceal obliteration faster and in fewer treatment sessions; (iii) endoscopic variceal ligation had a significantly lower rate of develop-**

**ment of portal gastropathy and rebleeding, (iv) while both techniques influenced gastric varices equally, there was significantly higher esophageal variceal recurrence after endoscopic variceal ligation than sclerotherapy.**

*Keywords:* Oesophageal varices, variceal band ligation

### PAPER DISCUSSION

Quite a few randomized trials and one meta-analysis [1] have compared endoscopic sclerotherapy with ligation for the treatment of bleeding esophageal varices. The consensus opinion is that banding ligation is associated with less complications, requires fewer treatment sessions to obliterate varices and probably reduces rebleeding and mortality of these patients. However, the vast majority of patients in these studies underwent elective endoscopic treatment. With the banding device loaded at the tip of the endoscope, the view is restriced and identification of the site of active bleeding might be difficult. At the moment, there are very few data on the success of banding ligation in controlling acute bleeding varices.

The study by Sarin *et al.*, compares endoscopic injection of absolute alcohol with banding ligation for esophageal varices. The authors are to be commended on the study design and careful documentation of clinical outcome parameters in the follow-up, which include rebleeding episodes, recurrence of varices, development of portal hypertensive gastropathy and gastric varices after successful obliteration of varices. In this study, only 12% of patients had active bleeding from esophageal varices and bleeding was successfully controlled in 80 – 85% by the two endoscopic treatments. Varices were eradicated after 4 sessions of banding ligation and 5 sessions of alcohol injection. No serious complication was reported except for esophageal structures in 5 patients receiving alcohol injections. The long-term results were remarkable. Very few rebleeding episodes have occurred and, in a mean follow-up of 8 months, the mortality rates were only 6.6 – 11.4%. However, it should be noted that 50% of patients in this study have Child's A class of liver disease and one-third of patients did not have cirrhosis. Patients with hepatic encephalopathy and renal impairment were excluded form the trial. Since hepatic reserve is the most important determinant of the clinical outcome in patients with variceal hemorrhage, this study only represents the results of the lower risk patients. When bleeding is active and endoscopic view is obscured, pharmacological therapy with a vasoactive agent as an adjuvant may improve the results of banding ligation in high risk patients [2].

In this study, patients receiving endoscopic banding ligation were found to have a higher recurrence of varices but a lower rebleeding rate compared to those receiving injection of alcohol. The authors attributed the low fequency of rebleeding to early obliteration of varices and fewer deep ulcers formed after banding ligation. Early recurrence of esophageal varices after banding ligation has also been reported by other workers [3]. Sarin proposed that the development of recurrent varices is a result of un-occluded perforator veins which allow communication of blood between the para-esophageal varices and the submucosal varices. Indeed, bu using computerized tomography, Lin *et al.*, showed that portal hypertensive patients with large para-esophageal varices are associated with higher rates of esophageal variceal recurrence and rebleeding after sclerotherapy [4]. We followed a cohort of patients after banding ligation by endosonography and have reported a similar observation [5]. Theoretically, if these perforators are sclerosed by injections at or around the esophago-gastric junction, recurrence of varices may be reduced. So far, clinical studies combining sclerotherapy with banding ligation have not improved the short-term results of banding ligation [6]. Whether the combined therapy could prevent recurrence of varices in the long run is still unknown.

While the safety of endoscopic banding ligation is largely undisputed, there is one major risk of this technique: esophageal perforation during insertion of the overtube. Perforation of the esophagus often occurs as a result of inability to relax the pharyngeal muscle by the patient and forceful insertion of the tube by the endoscopist. The development of multiple banding ligators (Speedband™ and Six-shooter™) in recent years has obviated the use of an overtube and is truly major advancement. Intubation is not more difficult with the endoscope loaded with these multiple band ligators and the procedure time is significantly shortened [7, 8]. Recently, a detachable mini-loop ligator has been developed and its use is currently under investigation.

I believe that endoscopic banding ligation will continue to gain popularity. Future development in this treatment modality should be directed to the prevention of recurrent varices formation, the improvement of multiple banding devices and the study of prophylactic banding ligation for selected cases with high risk of variceal bleeding.

### *References*

[1] Laine, L. and Cook, D. (1995). Endoscopic ligation compared with sclerotherapy for treatment of esophageal varices. A meta-analysis. *Ann. Intern. Med.*, **123**, 280-287.

[2] Sung, J. J. Y., Chung, S. C. S., Yung, M. Y., Lai, C. W., Lau, J. Y. W., Lee, Y. T., Leung, V. K. S., Li, M. K. K. and Li, A. K. C. (1995). Prospective randomized study of the effect of octreotide on rebleeding from esophageal varices after endoscopic ligation. *Lancet*, **346**, 1666-1669.

[3] Baroncini, D., Milandri, G. L., Borioni, D., Piemontese, A., Cennamo, V., Billi, P., Dal Monte, P. P. and D'lmperio, N. A. (1997). A prospective randomized trial of sclerotherapy versus ligation in the elective treatment of bleeding esophageal varices. *Endoscopy*, **29**, 235-240.

[4] Lin, C. Y., Lin, P. W., Tsai, H. M., Lin, X. Z., Chang, T. T. and Shin, J. S. (1994). Influence of para-esophageal venous collaterals on efficacy of endoscopic sclerotherapy for esophageal varices. *Hepatology*, **19**, 602-8.

[5] Leung, V. K. S., Sung, J. J. Y., Ahuja, A. T., Tumala, I. E., Lee, Y. T., Lau, J. Y. W. and Chung, S. C. S. (1997). Large para-esophageal varices on endosonography predict recurrence of esophageal varices and rebleeding. *Gastroenterology*, **112**, 1811-1816.

[6] Laine, L., Stein, G. and Sharma, V. (1996). Randomized comparision of ligation versus ligation plus sclerotherapy in patients with bleeding esophageal varices. *Gastroenterology*, **110**, 529-533.

[7] Saeed, A. Z. (1996). The Saeed Six-shoorter: a prospective study of a new endoscopic multiple rubber band ligator for the treatment of varices. *Endoscopy*, **28**, 559-564

[8] Sackmann, M. and Gerbes, A. L. (1996). Application of a multiple band ligator in active variceal bleeding. *Endoscopy*, **28**, 28-533.

Joseph J. Y. Sung M. D., PhD
Department of Medicine
Prince of Wales Hospital
The Chinese University of Hong Kong
Shatin, Hong Kong
China

### Leak after Whipple Resection: Preventable?

### ABSTRACT

*Howard, J. M. (1997) Pancreatojejunostomy: Leakage is a preventable complication of the whipple resection. Journal of the American College of Surgeons;* **184**, *454-457.*

***Background:*** **Leakage of the pancreaticojejunal anastomosis has been a major complication after pancreaticoduodenectomy (Whipple operation), frequently reported in an incidence of 5 percent to 15 percent. The most widely used techniques of anastomosis have been variations of end-to-end pancreaticojejunostomy. Complicating 152 end-to-end anastomoses, done by me (including 98 for carcinoma of the pancreas or ampulla), were 5 pancreatic anastomotic leaks; the fifth patient died of this complication.**

***Study Design:*** **The death resulting from a pancreatic anastomotic fistula led me to change my technique to an end of the pancreas to side of the jejunum, mucosa-to-mucosa, pancreaticojejunostomy (intubated), a modification of the technique described by Cattell and used since**

